# Medical treatment of early stage and rare histological variants of epithelial ovarian cancer

**DOI:** 10.3332/ecancer.2015.584

**Published:** 2015-10-22

**Authors:** Nicoletta Tomasi Cont, Annamaria Ferrero, Fedro Alessandro Peccatori, Marta D’Alonzo, Giovanni Codacci-Pisanelli, Nicoletta Colombo, Nicoletta Biglia

**Affiliations:** 1 Academic Division of Gynaecology and Obstetrics, Mauriziano Hospital, University of Turin, Italy; 2 Fertility and Pregnancy Unit, Medical Gynaecologic Oncology Division, European Institute of Oncology, Milan, Italy; 3 Department of Medical and Surgical Science and Biotechnology, University of Rome ‘La Sapienza’, Italy; 4 Medical Gynaecologic Oncology Division, European Institute of Oncology, University of Milan-Bicocca, Italy

**Keywords:** ovarian neoplasm, biological markers, personalised medicine

## Abstract

Epithelial ovarian cancer is often considered a single pathological entity, but increasing evidence suggests that it is rather a group of different neoplasms, each with unique pathological characteristics, molecular features, and clinical behaviours. This heterogeneity accounts for the different sensitivity to antineoplastic drugs and makes the treatment of ovarian tumours a challenge.

For early-stage disease, as well as for heavily pre-treated patients with recurrent ovarian cancer, the benefit of chemotherapy remains uncertain.

Clear-cell, mucinous, low-grade serous, and endometrioid carcinomas show different molecular characteristics, which require different therapeutic approaches. In the era of personalised cancer medicine, understanding the pathogenesis and the genetic background of each subtype of epithelial ovarian tumour may lead to a tailored therapy, maximising the benefits of specific treatments and possibly reducing the side effects. Furthermore, personal factors, such as the patient’s performance status, should be taken into account in the management of ovarian cancer, with the aim of safeguarding the patients’ quality of life.

## Introduction

Ovarian cancer is the sixth most common cancer diagnosed among women and the leading cause of death from gynaecologic malignancies worldwide [1].

Ovarian neoplasms include epithelial ovarian cancer, which represents about 90% of all ovarian tumours, and non-epithelial ovarian cancer, including stromal and germ cell tumours. Morphologically, epithelial ovarian cancer is classified into five main histologic subtypes: high-grade serous, which accounts for 70% of all epithelial cancer, low-grade serous, endometrioid, mucinous, and clear-cell tumours [2]. These different ovarian cancer subtypes show a distinct mutational spectrum: high-grade serous ovarian cancers present mutations of TP53 in about 96% of cases, and they are also characterised by BRCA 1/2 mutations (including a combination of germline and somatic mutations) in 20% of cases, [3]. Low-grade serous ovarian cancer is often associated with k-ras, b-raf, and ERBB2 mutations, while TP53 is rarely mutated [4]. Clear-cell ovarian cancers have less frequent TP53 mutations but have recurrent ARID1A and PIK3CA mutations. Endometrioid ovarian carcinomas have a similar pattern of genetic aberrations, with low rate of TP53 mutations and prevalent ARID1A, PIK3CA, and CTTNB1 mutations. Mucinous ovarian tumours show KRAS mutations [3]. These genetic characteristics likely reflect a distinct pathogenesis and lead to different biological behaviour, with impact on prognosis and on response to antineoplastic treatments.

On the basis of molecular pathogenesis, it is possible to divide epithelial ovarian tumours into two subtypes. Type-1 ovarian cancers tend to be low grade and to have an indolent biological behaviour, with characteristic genetic mutations. It is hypothesised that type-1 ovarian tumours evolve from benign lesions, including endometriosis and borderline lesions. Actually, the same genetic mutations have been recognised in endometrioid ovarian cancer and concomitant endometriosis. Type-1 category includes low-grade serous, endometrioid, mucinous and clear-cell ovarian carcinoma [4]. On the contrary, type-2 ovarian tumours are high grade, aggressive tumours. Mutations involve different genes and it is hypothesised that these tumours originate from the fimbriae of the fallopian tubes as intraepithelial tubal carcinoma, which subsequently metastasise to the ovaries and to the peritoneal surface. Type-2 cancers include high-grade serous, high-grade endometrioid, malignant mixed mesodermal, and undifferentiated ovarian tumours. These subtypes differ not only for the genetic mutations and the precursor lesions: they show different prognosis, patterns of spread and response to chemotherapy [4].

Diagnosis of type-I ovarian cancer often occurs at an early stage, when the neoplasm is confined to one or both ovaries. These tumours are genetically stable: each histological subtype presents a typical molecular profile and specific cell-signalling pathways that might become a target for new therapies [5]. Type-II ovarian cancers are generally diagnosed at advanced stage and have an unstable genome. The high frequency of homologous recombination defects shown by these tumours represents a possible Achille’s heel and treatment with PARP inhibitors demonstrated a significant antitumoural activity [5].

Although notable efforts have been made during the years, the mortality of patients with ovarian cancer remains high, particularly for those with advanced disease. Treatment guidelines do not differentiate among histology or molecular subtypes: one size still fits all and stage is the only discriminator for treatment [6, 7, 8]. Nonetheless, ovarian cancer is not a single disease, rather a set of subtypes, each with a unique molecular profile and biological behaviour with relevant therapeutic implications.

The purpose of this article is to discuss treatment individualisation according to stage and histological subtypes. Moreover, a section is dedicated to hormonal treatment of epithelial ovarian cancer.

## Early-stage ovarian cancer: When and what type of adjuvant chemotherapy is indicated?

Early-stage ovarian cancer includes FIGO Stage Ia, Ib, and Ic (Table 1) [9]. The prognosis of early-stage ovarian cancer is good, with a five-year survival rate of 70–90% [8].

Tumour removal and adequate surgical staging, followed in most cases by adjuvant chemotherapy, represent the primary treatment for early-stage ovarian cancer [6]. Nonetheless, despite adequate frontline treatment, the risk of relapse is not negligible. The rationale of adjuvant chemotherapy after complete removal of the disease and adequate surgical staging is to eradicate any residual microscopic deposits of cancer cells responsible of potential recurrence of disease. Even if a number of evidences point to an advantage of adjuvant chemotherapy in early-stage ovarian cancer, the optimal regimen, and duration is still debated.

A Cochrane meta-analysis assessed the survival advantage of adjuvant chemotherapy in early-stage ovarian cancer [10]. Five randomised clinical trials were included in the analysis. The earliest three trials recruited a small number of patients and lacked enough events to demonstrate a possible impact of adjuvant treatment. On the contrary, the ICON1 and the ACTION trial [11] included a larger number of patients and had a sufficient power to demonstrate a treatment effect. These two trials randomised patients with serous, mucinous, endometrioid, clear-cell, and undifferentiated ovarian carcinomas to receive platinum-based chemotherapy or no chemotherapy [11]. The five-year and the updated 10-year overall survival rate was significantly better for women receiving adjuvant chemotherapy compared with the control group. Similar findings were reported for progression-free survival [10].

Additionally, these two trials address the question of which patients with early-stage ovarian cancer benefitted most from adjuvant chemotherapy. The ICON1 trial stratified patients in low/medium risk (FIGO-stage Ia G1-G2 or FIGO-stage Ib/Ic G1) and high risk (FIGO-stage Ia G3, Ib/Ic G2-G3, any clear cell). Both overall and progression-free survival were better in high-risk patients, but no difference was observed among treated and non-treated low–medium risk patients at five- and 10-year follow-up [12]. One of the limitations of this study is represented by an unclear indication for surgical staging, resulting in suboptimally staged patients. Conversely, the ACTION trial strongly recommended a complete surgical staging and planned a subgroup analysis on suboptimally and optimally staged patients. Patients with FIGO Stage-Ia/Ib G2–G3, FIGO Stage-Ic/IIa were included. Among the suboptimally staged women, adjuvant chemotherapy increased the overall and disease-free survival, whereas in the optimally staged patients, no difference was observed between the treated group and the control group. At a median follow-up of 10 years earlier, data were confirmed [10]. These results suggested that there is a subgroup of patients at good prognosis that apparently do not benefit from adjuvant chemotherapy. In particular, it is hypothesised that chemotherapy impacts only on occult disease in suboptimally staged patients. Nonetheless, a benefit in optimally staged tumour cannot be excluded.

In summary, adjuvant chemotherapy may be avoided for low-risk, optimally staged, Stage-I patients (FIGO Stage-Ia/Ib, G1–G2); chemotherapy is indicated after surgery for patients with high-risk Stage-I disease (FIGO Stage-Ic, G3). In case of suboptimal surgical staging of low-risk Stage-I patients, benefits and effect of adjuvant chemotherapy should be discussed with each individual patient [6, 7, 10].

No sound evidences that a combination of carboplatin plus paclitaxel for early-stage disease is superior to carboplatin alone [13] are available. Studies that compare these two regimens are lacking [8]. Indirect suggestions of greater efficacy when combining carboplatin and taxanes are gathered by results in advanced stage disease. A well-designed trial is needed to identify the optimal chemotherapy regimen in this patient population. In the absence of clear recommendations, single-agent carboplatin seems a reasonable option for intermediate and high-risk early-stage ovarian cancer patients [13].

The optimal duration of adjuvant chemotherapy remains a matter of investigation. A randomised trial which compared three versus six cycles of platinum plus paclitaxel for early-stage ovarian cancer, revealed a 24% reduction in recurrence rate in patients who underwent six courses of chemotherapy [14]. A subgroup analysis stratifying patients on the basis of clinical and pathological features, showed a statistically significant benefit of six versus three cycles of chemotherapy only in serous tumours, while outcome for non-serous tumours was not influenced by the duration of chemotherapy [15]. Again, there may be a subgroup of patients who do not benefit from intensive adjuvant chemotherapy and future research is needed to confirm these hypotheses.

## Clear cell, low-grade serous, mucinous or endometrioid ovarian cancers: Different therapeutic approaches for different neoplasms

The landscape of medical treatment of ovarian cancer has changed in the last years, thanks to improved biologic understanding. Beyond traditional chemotherapy, several target agents has been tested with the aim of improve outcome of patients with ovarian cancer. Angiogenesis play a role in promoting tumour growth and favouring metastasis. The antiangiogenetic agent bevacizumab improve progression-free survival in advanced stage and recurrent ovarian cancer, highlighting the importance of angiogenesis pathway in the tumour development. Phase-III studies are testing the efficacy of tyrosine kinase inhibitors, which target pro-angiogenesis proteins [16]. Promising results are obtained by antiangiogenetic therapy in epithelial ovarian cancers, independently by histological features. But molecular and genomic understanding suggests that key advancement in ovarian cancer treatment may be kept into the different spectrum of genomic aberrations that each ovarian cancer subtype displays.

## Clear-cell carcinoma

Ovarian clear-cell carcinoma accounts for 10% of all epithelial ovarian cancers [2]. It tends to present as an adnexal mass and diagnosis occurs at an earlier stage than other subtypes of epithelial ovarian cancer (Stage I–II in 57–81% of cases) [17]. As endometrioid tumours, it has been associated with endometriosis [18]. Genetic analysis revealed high frequency of PI3K and ARID1A mutations, whereas p53 is generally wild type [17]. Treatment plan is the same as for other epithelial ovarian cancers, including surgical cytoreduction and platinum-based chemotherapy, but some studies suggest that response to standard carboplatin-paclitaxel regimen may be low.

Early-stage clear-cell disease shows a better prognosis than serous subtype. It has been reported that 10-year survival rate is 87% for patients with FIGO Stage-Ia and FIGO Stage-Ib clear-cell carcinoma, compared with 68% for patients with serous ovarian cancer in a similar stage [19]. Guidelines recommend adjuvant chemotherapy for clear-cell ovarian tumours, independently of stage. The ACTION trial reported similar disease-free survival for patients with early-stage clear-cell carcinoma treated or not with chemotherapy after surgery [11, 20]. On the basis of this suggestion, two analyses were conducted and simultaneously published in 2012 [21, 22]. These studies investigated the benefit of chemotherapy after adequate surgery for early-stage clear-cell carcinoma. Both groups reported that chemotherapy after complete surgical staging for Stage-Ia clear-cell carcinoma did not influence disease-free survival, and the impact on overall survival did not reach statistical significance. These preliminary results suggest that for optimally staged FIGO Ia-Ib disease, adjuvant platinum-based chemotherapy might not confer survival advantage. Further studies are needed to confirm the possibility that selected patients with early-stage clear-cell carcinoma could avoid chemotherapy after complete surgery.

In advanced disease, different elements should be taken into consideration. It was suggested that the worse prognosis of advanced stage clear-cell carcinoma compared with high-grade serous cancer, could be due to the reduced sensitivity to standard carboplatin plus paclitaxel-based chemotherapy [17]. The need of improving prognosis of clear-cell cancer patients prompted the development of new treatment strategies: in the last years, many studies were conducted in order to evaluate the effectiveness of different antineoplastic agents in this particular neoplasm. Preclinical models showed a high response of clear-cell cancer to trabectedin [23]. Kawano *et al* tested the combination of trabectedin and irinotecan/topotecan on human ovarian clear-cell cancer lines, showing synergism between these antineoplastic agents, which interact with DNA via different mechanisms [24].

On the basis of these promising results, some authors reported the potential therapeutic benefit of cisplatin and irinotecan in treating ovarian clear-cell cancer [25] and the JGOG group conducted a randomised Phase-III trial which compared the efficacy and safety of irinotecan plus cisplatin versus paclitaxel plus carboplatin. Data of the JGOG3017/GCIG trial are not published yet, but preliminary results were presented at ASCO in 2014. The two-year progression-free survival of cisplatin–irinotecan arm is 73% compared with 77.6% of carboplatin–paclitaxel arm, with a hazard ratio of 1.171 (0.867–1.580). Similar results were reported for the two-year overall survival with 85.5% in cisplatin–irinotecan arm versus 87.4% in carboplatin–paclitaxel arm (HR 1.133 (0.796–1.613)).

The unique molecular and genomic profile of clear-cell carcinoma encourages therapeutic innovations and opens the way to targeted therapies. In 30–40% of cases, clear-cell carcinoma shows activating mutations of the PI3KCA gene [17]. The PI3K–AKT–mTOR pathway is a potential target for treatment [26]. In the previously cited studies [24], the association of the mTOR inhibitor everolimus with trabectedin alone or with irinotecan reduced the onset of chemoresistance and enhanced the antitumor efficacy. On the basis of these results, the gynaecologic oncology group has initiated a Phase-II evaluation of temsirolimus in combination with carboplatin and paclitaxel, followed by temsirolimus consolidation as first-line therapy for patients with Stage-III–IV clear-cell carcinoma (GOG 268 trial).

## Low-grade serous carcinoma

The identification of low-grade serous ovarian carcinoma (LGSOC) as a different histological type has only recently been accepted and its biological and clinical characteristics are not yet completely described.

From a histological and molecular point of view, LGSOC appears to be strictly connected with tumours of low malignant potential that arises from the ovary or from the peritoneum. Among genetic anomalies, it may be worth mentioning that LGSOC show a high frequency of mutations affecting K-RAS or B-RAF [27]. Expression of mitogen-activated protein kinases (MAPK) is generally high, while p53 mutations are less common than in HGSOC.

LGSOC frequently affects younger women that are diagnosed with locally advanced disease. These patients are generally treated like HGSOC, still all evidence suggests that they receive only limited benefit from platinum chemotherapy and PFS is relatively short. Nonetheless, due to the indolent nature of this disease, OS is generally longer than that of HGSOC: patients undergo multiple lines of medical treatment and surgical resections [28], but this disease is invariably fatal.

Even if LGSOC was only recently identified as a specific entity, its resistance to standard medical treatment and the presence of well-described molecular anomalies have stimulated the evaluation of target agents in this molecular subtype of ovarian cancer. Results, particularly with MEK inhibitors, are promising [29] and this line of research is being actively evaluated.

## High-grade serous ovarian cancer: BRCA 1/2, homologous recombination defect and parp inhibitors

About 20% of epithelial ovarian tumours have BRCA 1/2 germline or somatic mutations [30]. Mutated patients often show high response to platinum-based chemotherapy, long disease-free interval and improved overall survival. High-grade serous ovarian carcinoma is the main histological subtype [30].

BRCA 1/2 are involved in homologous recombination repair of double-strand DNA breaks. BRCA1/2-deficient cells develop a predisposition to aberration in chromosome structure, which lead to genomic instability and subsequent cancer susceptibility [31]. Others genes are involved in DNA repair pathway, including EMSY, PTEN, RAD51C, ATM/ATR, and contribute to create the phenotype or BRCAness, a pattern of biological and clinical behaviour that some sporadic ovarian cancers share with hereditary BRCA cancers. The cancer genome atlas suggests that defects in homologous recombination are present in up to half of high-grade serous ovarian tumours [3].

Homologous recombination defects become a crucial target for a new class of antineoplastic drug known as PARP inhibitors. Poly (ADPribose) polymerase is a family of enzymes involved in the pathway of single-strand DNA breaks. In a cell where PARP is inhibited, the single-strand DNA break is converted into a double-strand DNA break. If the same cell has a defective homologous recombination, as in BRCA-deficient cancer cells, the combination of these dysfunctions lead to synthetically lethal death [32].

Preclinical studies demonstrated the activity of PARP inhibitors in BRCA-deficient cells [32]. Phase-II trial confirmed that PARP inhibitors improve progression-free survival, without an effect on overall survival [33]. A subgroup analysis highlighted that these encouraging results are enhanced in BRCA 1/2-mutated ovarian cancers [33]. The ongoing Phase-III trials are testing the role of olaparib as maintenance treatment after chemotherapy for relapsed ovarian cancer but also for newly diagnosed, advanced ovarian tumours. Furthermore, the potential efficacy of others PARP inhibitors is currently being evaluated, both in BRCA mutated and sporadic ovarian cancer patients.

These promising data led to the approval by the FDA of the PARP inhibitor olaparib as maintenance therapy for BRCA 1/2 mutated ovarian cancer patients who have received three or more lines of prior chemotherapy. This is the first personalised therapy approved for ovarian cancer [32].

## Mucinous carcinoma

Mucinous tumours account for approximately 10–15% of all ovarian tumours, but most are benign or borderline. Only 3–4% of invasive ovarian cancers are mucinous [2]. Clinically, they arise as large multiloculated cysts with mucus-containing fluid. Molecular studies suggest that mucinous ovarian tumours are different from serous, with a higher rate of KRAS and HER2 mutations whereas p53 mutations are less frequent [34].

About 83% of mucinous ovarian cancers are diagnosed at Stage I and are usually well-differentiated [35]. Early-stage, well-differentiated mucinous ovarian carcinoma have good prognosis with a five-year survival rate of 90%. Generally, they are characterised by an expansive growth pattern and can resemble tumours arising from the gastrointestinal tract [36]. In ACTION and ICON1 trials, 19.5% of patients had mucinous ovarian cancer. In this subgroup, there was no statistically significant difference in outcome between treated and not treated patients, suggesting the absence of benefit due to chemotherapy [34]. These data, in association with the good outcome for early-stage low-grade mucinous ovarian tumours, question if adjuvant chemotherapy is really necessary for such cases.

Advanced-stage mucinous tumours display an infiltrative growth pattern and a relative platinum resistance with reduced response rates to standard carboplatin plus paclitaxel-based chemotherapy and worse disease-free survival and overall survival than serous ovarian cancer [36]. Given the similarities between mucinous ovarian neoplasm and gastrointestinal (GI) mucinous tumours, preclinical and clinical studies tested some GI effective antineoplastic agents such as oxaliplatin, 5-fluorouracil, capecitabine, and others, in a setting of untreated or recurrent patients with mucinous ovarian cancer [36]. The ongoing mEOC trial is a GCIG randomised Phase-III study, which compares carboplatin and paclitaxel ± bevacizumab with oxaliplatin and capecitabine ± bevacizumab as first-line chemotherapy in patients with mucinous epithelial ovarian cancer. Aims of the study are to evaluate whether oxaliplatin plus capecitabine is superior to standard regimen and to confirm the benefit of adding bevacizumab in the treatment of these particular tumours.

Genomic studies reported that human epidermal growth factor receptor 2 (HER2) gene is amplified and its protein overexpressed in 18–35% mucinous ovarian cancers [37]. It is not clear what is the prognostic value of HER2 in mucinous ovarian tumours, but some authors proposed anti-HER2 target therapy, with sporadic encouraging results [38]. However, reports are conflicting and trials evaluating the role of trastuzumab and others anti-HER2 compounds are needed. Also, KRAS mutation is frequent in mucinous ovarian tumours, representing another therapeutic option. Recently, a multicentric study analysed HER2 and KRAS profile in a large cohort of mucinous cancers [39]. Authors proposed an algorithm based on HER2 and KRAS testing with subsequently target therapies for chemoresistant mucinous tumours, which may be tested in future researches.

## Endometrioid carcinoma

Endometrioid epithelial ovarian tumours represent 10% of all ovarian cancer. They often occur in younger women than other subtypes and are mostly diagnosed at early stage with good outcome [2]. As clear-cell carcinoma, an association with endometriosis has been recognised, a feature that confers better prognosis with lower rates of recurrence than serous tumours [40]. A prospective study was conducted by Storey on 270 patients with endometrioid ovarian carcinoma [41]. Analysing data from Stage-I patients, no statistically significant difference in progression-free survival and overall survival was found between endometrioid and serous tumours. Similarly, no difference in response rates to platinum-based chemotherapy was observed between endometrioid and serous tumours [41]. These findings suggest that, despite different molecular features, the same therapeutic approach is recommended for endometrioid and serous epithelial ovarian cancer, both for early-stage and advanced-stage tumours.

Advanced disease frequently responds to first-line platinum–taxane combination chemotherapy, but the relapse rate is high and associated with poor prognosis. In an attempt to overcome chemoresistance, studies investigated the role of target therapy. Molecular analysis demonstrated dysregulation of the Wnt/β-catenin/Tcf and PI3K/AKT/mTOR signalling [2]. *In vitro* and *in vivo* models suggested the efficacy of drugs that regulate these mutated pathways, and further applications may be explored [42, 43].

The main characteristics of these three histologic subtypes of ovarian cancer are compared in Table 2.

## Role of hormonal therapy in ovarian cancer

Several studies investigated the role of hormones on ovarian carcinogenesis. *In vitro* and *in vivo* studies showed that a subset of ovarian cancer cells is susceptible to oestrogens, with increased cell proliferation and tumour growth [44]. Moreover, antioestrogens can inhibit ovarian cancer cells proliferation in cell cultures and xenograft models [45]. Only a subgroup of tumours responds to hormonal therapy and it was supposed a relationship between response rate and oestrogen receptors (ER) expression. A review reported that about 67% of ovarian cancer expressed ER [46]. There are some evidences about the prognostic relevance of ER expression. A large study including almost 600 patients showed that ER-positive (ER expression > 10%) ovarian tumours have better outcome with higher disease-specific survival than ER-negative tumours [47].

There are two types of ER: ER-α and ER-β. Previous studies suggested that ER-β is predominant in normal ovaries, with an antiproliferative activity. Conversely, ER-α is highly expressed in ovarian cancer cells and stimulate proliferation. It is possible that in ovarian tumours the balance between ER-α and ER-β is altered [48]. The result is an oestrogen-induced tumour growth.

Preclinical and clinical studies evaluated the impact of antioestrogen therapy, such as tamoxifen and oestrogen deprivation, due to aromatase inhibitors on ovarian cancer progression.

Tamoxifen inhibits ovarian cancer-cell proliferation *in vitro* [49]. On the basis of these results, clinical studies tested the efficacy of tamoxifen for patients with recurrent ovarian cancer. A review of 20 studies demonstrate an overall response rate of 13%, with 4% of complete response and 9% of partial response. In addition, 35% of patients have stable disease [48]. It should be noted at least two major bias in the previous cited studies that could underestimate the efficacy of ER-blockage. First, the lack of trials specifically designed to test the influence of estrogen receptor status on response to tamoxifen. Most studies did not report the oestrogen receptor status of the tumour. On the other hand, the largest series published by Hatch *et al* showed higher response to tamoxifen in ER-positive patients than in ER-negative patients [50]. Another limit is related to the absence of well-defined target population. In many cases, patients were heavily pretreated and often their tumour had become chemoresistant. Higher response to hormonal therapy was observed in patients treated with tamoxifen after no more than one previous line of chemotherapy, with an overall response rate of 25%, including 8.8% of complete response [49].

Similar results are obtained by oestrogen deprivation induced by aromatase inhibitors (AIs) [48]. Some data suggest that there is a local oestrogenic synthesis related to an ovarian aromatase. These intratumoral oestrogens stimulate ovarian cancer cells proliferation independently of circulating hormones [51]. Clinical studies evaluated the role of third-generation AIs (letrozole, anastrozole, and exemestane) in recurrent ovarian cancer, demonstrating complete response in 1% of patients, partial response in 7% and stable disease in 33% [48]. Again, many studies did not select patients on the basis of ER status. A study demonstrated higher response to letrozole in ER-positive, recurrent ovarian cancer, with a rate of CA125 response rate of 17% and a rate of stable disease of 36% [52], an efficacy comparable to salvage chemotherapy, but less toxic for the patients.

The hormonal mitogenic effect involves oestrogens, ERs and the oestrogen-regulated genes. Other molecules, such as PI3K/mTOR, are activated by oestrogens [51]. Studying these pathways may lead to the development of therapies that combine target agents and oestrogen inhibitors in order to potentiate the antitumour effect.

It is debated if there is a role for hormonal therapy in patients with recurrent ovarian cancer, with the aim of prolonging the platinum-free interval: at present no clear data support this use and future research is needed.

## Conclusion

Epithelial ovarian cancer is not a single disease, but a heterogeneous group of neoplasms. Classification on the basis of morphological, immunohystochemical, and genetic analysis and progresses in understanding the molecular background of carcinogenesis contributes to identify specific and unique tumours subtypes with different behaviour, prognosis, and response to chemotherapy.

The current indications for the medical treatment of epithelial ovarian cancer are the same, independently from histological subtypes. It is hoped that the current one size-for-all chemotherapy regimens will evolve into genetic-specific treatments based on molecular markers.

## Declaration of interest

The authors declare that they have no conflict of interest.

## Figures and Tables

**Table 1.** 2014 FIGO ovarian, fallopian tube, and peritoneal cancer staging system and corresponding TNM.

**I**	**Tumour confined to ovaries or fallopian tube(s)**	**T1**
IA	Tumour limited to one ovary (capsule intact) or fallopian tube. No tumour on ovarian or fallopian tube surface. No malignant cells in the ascites or peritoneal washings.	T1a
IB	Tumour limited to both ovaries (capsules intact) or fallopian tubes. No tumour on ovarian or fallopian tube surface. No malignant cells in the ascites or peritoneal washings	T1b
IC	Tumour limited to one or both ovaries or fallopian tubes, with any of the following:	T1c
IC1	Surgical spill intraoperatively	
IC2	Capsule ruptured before surgery or tumour on ovarian or fallopian tube surface	
IC3	Malignant cells present in the ascites or peritoneal washings	
**II**	**Tumour involves one or both ovaries or fallopian tubes with pelvic extension (below pelvic brim) or peritoneal cancer (Tp)**	**T2**
IIA	Extension and/or implants on the uterus and/or fallopian tubes/and/or ovaries	T2a
IIB	Extension to other pelvic intraperitoneal tissues	T2b
**III**	**Tumour involves one or both ovaries, or fallopian tubes, or primary peritoneal cancer, with cytologically or histologically confirmed spread to the peritoneum outside the pelvis and/or metastasis to the retroperitoneal lymph nodes**	**T3**
IIIA	Metastasis to the retroperitoneal lymph nodes with or without microscopic peritoneal involvement beyond the pelvis Positive retroperitoneal lymph nodes only (cytologically or histologically proven)	T1, T2, T3aN1
IIIA1	Metastasis ≤ 10 mm in greatest dimension (note this is tumour dimension and not lymph node dimension) Metastasis *N* 10 mm in greatest dimension	
IIIA1(i)	Microscopic extrapelvic (above the pelvic brim) peritoneal involvement with or without positive retroperitoneal lymph nodes	T3a/T3aN1
IIIA1(ii)		
IIIA 2	Macroscopic peritoneal metastases beyond the pelvic brim ≤ 2 cm in greatest dimension, with or without metastasis to the retroperitoneal lymph nodes	T3a/T3aN1
IIIB		T3b/T3bN1
III C	Macroscopic peritoneal metastases beyond the pelvic brim *N* 2 cm in greatest dimension, with or without metastases to the retroperitoneal nodes (Note 1)	T3c/T3cN1
**IV**	**Distant metastasis excluding peritoneal metastases**	**Any T, Any *N*, M1**
Stage IV A	Pleural effusion with positive cytology	Any T, Any *N*, M1
Stage IV B	Metastases to extra-abdominal organs (including inguinal lymph nodes and lymph nodes outside of abdominal cavity) (Note 2)	

Based on ‘DG Mutch and J Prat. 2014 FIGO staging for ovarian, fallopian tube and peritoneal cancer. Gynecologic Oncology 2014;133:401-04’ **Notes:** 1. Includes extension of tumour to the capsule of liver and spleen without parenchymal involvement of either organ. 2. Parenchymal metastases are Stage IV B.

**Table 2.** Characteristics of clear cell, mucinous, and endometrioid ovarian carcinoma in comparison.

	Clear-cell carcinoma	Mucinous carcinoma	Endometrioid carcinoma
Relative frequency	10% of all EOC	3% of all EOC	10% of all EOC
Clinical features	Adnexal mass	Large unilateral cyst with mucus-containing fluid	Unilateral cyst
Precursor lesions	Endometriosis	Unknown (cystoadenoma and mucinous borderline tumour?)	Endometriosis and borderline adenofibroma
Diagnosis	Generally at early stage	Generally at early stage	Generally at early stage
Prognosis	Intermediate (relatively good prognosis for early stage, poor prognosis for advanced stage)	Favourable	Favourable
Genetic mutations	PI3K, ARID1A	KRAS, HER2	PTEN, β-catenin, PI3K
Platinum sensitivity	Low	Low	High
New therapeutic agents	Trabectedin irinotecan/topotecan, cisplatin	Oxaliplatin, capecitabine, 5-fluorouracil,	None
